# Supplementary figures and images for: From Abstainers to Dependent Drinkers: alcohol consumption patterns and risk factors among Portuguese university students
Source: PeerJ. 2025 Sep 24;13:e20026. doi: 10.7717/peerj.20026 (PMC12476170; doi:10.7717/peerj.20026)

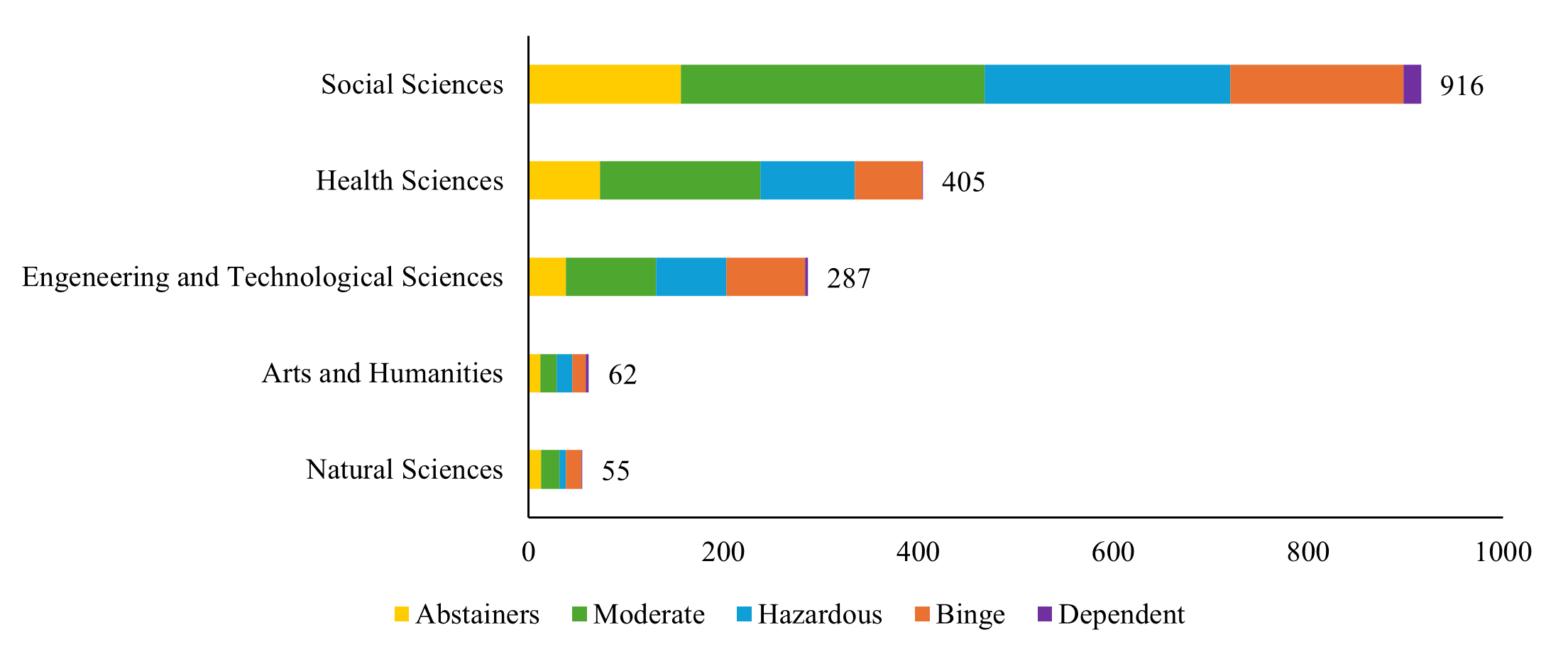

Supplement: Supplemental Information 1 [file peerj-13-20026-s001.png]

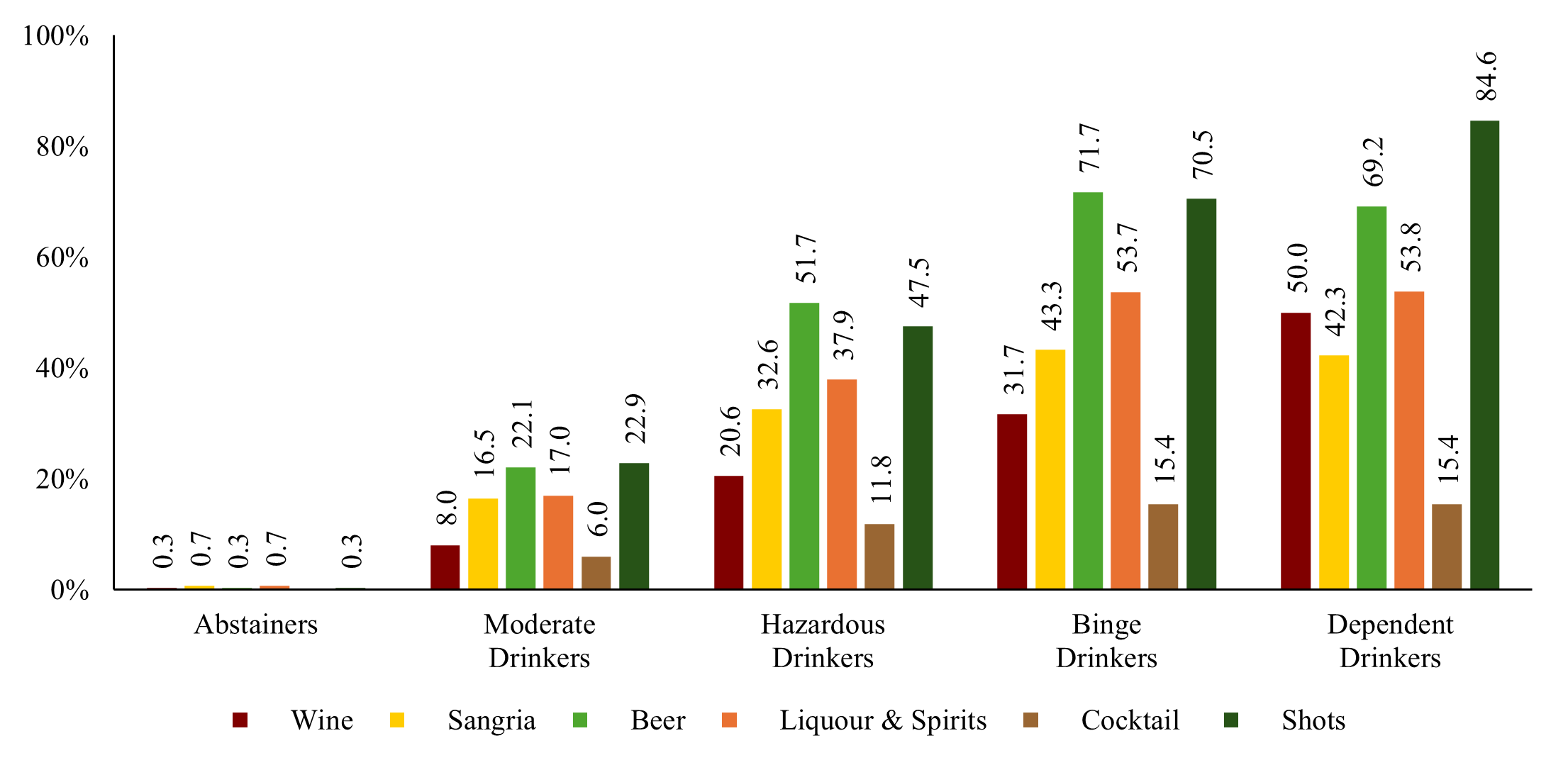

Supplement: Supplemental Information 2 [file peerj-13-20026-s002.png]

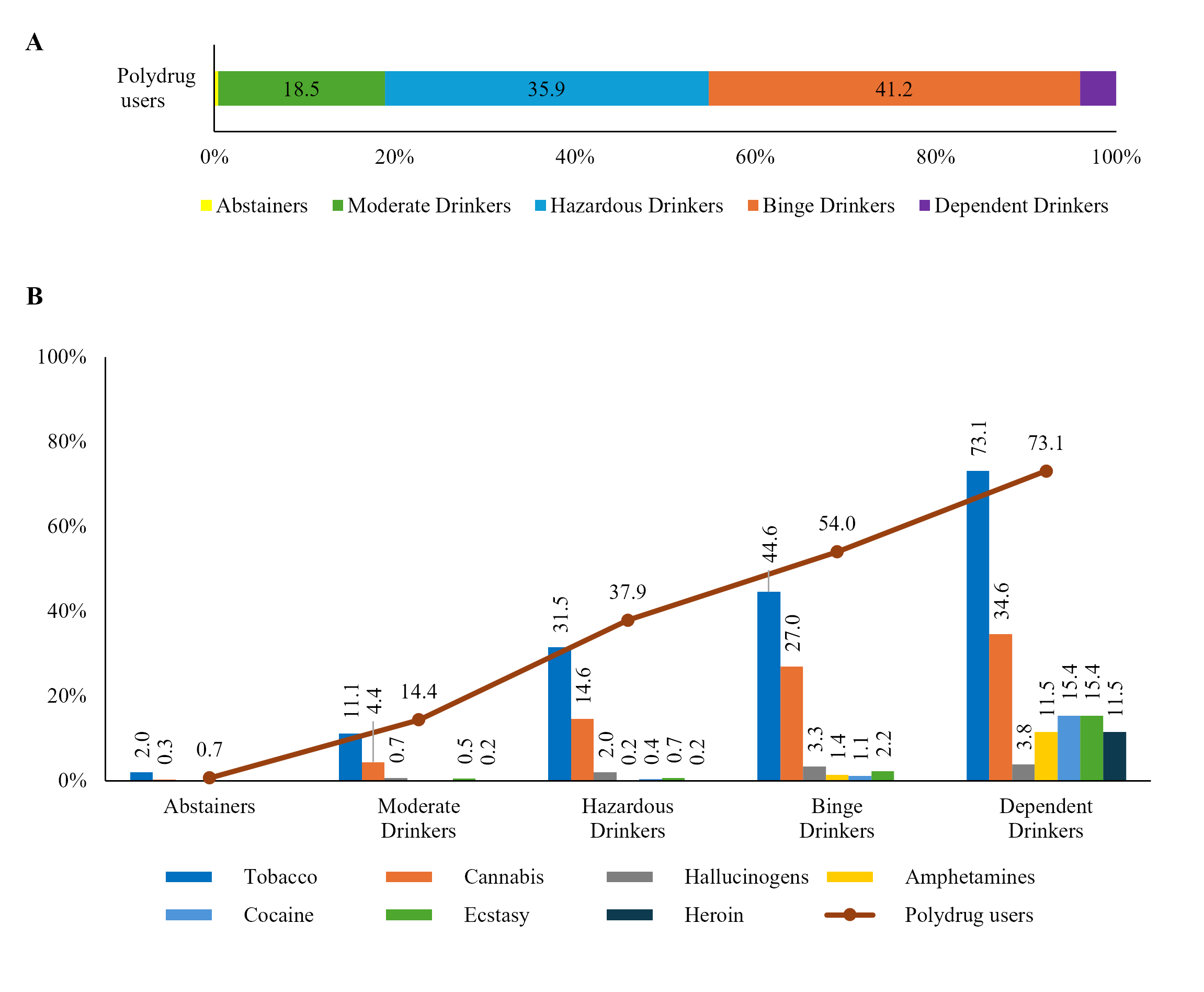

Supplement: Supplemental Information 3 — (A) Distribution of Drinking Groups within polydrug users; Of all polydrug users, 0.4% were Abstainers, 18.5% Moderate Drinkers, 35.9% Hazardous Drinkers, 41.2% Binge Drinkers, and 4.0% Dependent Drinkers. (B) Prevalence of single-substance and polydrug use across the Drinking Groups; Grouped bar showing the percentage of use, within Drinking Groups, for tobacco, cannabis, cocaine, ecstasy, hallucinogens, amphetamines, and heroin. The overlaid line indicates the percentage of polydrug use across the Drinking Groups. [file peerj-13-20026-s003.png]
